# Broadband Plasmonic Metamaterial Optical Absorber for the Visible to Near-Infrared Region

**DOI:** 10.3390/nano13040626

**Published:** 2023-02-04

**Authors:** Ahmad Musa, Touhidul Alam, Mohammad Tariqul Islam, Mohammad Lutful Hakim, Hatem Rmili, Ahmed S. Alshammari, Md. Shabiul Islam, Mohamed S. Soliman

**Affiliations:** 1 Pusat Sains Angkasa (ANGKASA), Institut Perubahan Iklim, Universiti Kebangsaan Malaysia, Bangi 43600, Selangor, Malaysia; 2 Department of Electrical, Electronic and Systems Engineering, Faculty of Engineering and Built Environment, Universiti Kebangsaan Malaysia, Bangi 43600, Selangor, Malaysia; 3 K. A. CARE Energy Research and Innovation Center, King Abdulaziz University, Jeddah 21589, Saudi Arabia; 4 Electrical and Computer Engineering Department, Faculty of Engineering, King Abdulaziz University, Jeddah 21589, Saudi Arabia; 5 Department of Electrical Engineering, College of Engineering, University of Ha’il, Ha’il 81481, Saudi Arabia; 6 Faculty of Engineering (FOE), Multimedia University, Persiaran Multimedia, Cyberjaya 63100, Selangor, Malaysia; 7 Department of Electrical Engineering, College of Engineering, Taif University, P.O. Box 11099, Taif 21944, Saudi Arabia; 8 Department of Electrical Engineering, Faculty of Energy Engineering, Aswan University, Aswan 81528, Egypt

**Keywords:** metamaterial absorber, nanoarchitecture, optical window, infrared window, photon conversion, energy harvesting

## Abstract

An oblique angle and polarization insensitive metamaterial absorber (MA) are highly desired for the visible and infrared optical applications like, wave energy harvesting, optical filters, and detecting thermal leaks and electrical defects. In this paper, a multi-layered MA consisting of two layers of tungsten resonators on a silicon dioxide substrate, coated with additional SiO_2_ materials is investigated. The unit cell size of the MA is 0.5λ × 0.5λ × 0.8λ, at the lowest wavelength. The proposed MA offers an average absorption of 92% from 400 nm to 2400 nm with stable oblique incident angles up to 45°. The structure also achieves polarization insensitivity at the entire visible and near-infrared spectrum. Moreover, the MA is found highly compatible for solar absorber applications with high y A_AM1.5_. The structure is also compatible for filter application in optical communication system by modifying the plasmonic nano structure. The modified structure can block the wavelengths of the visible band (450 nm to 800 nm) and transmit optical communication bands (800 to 1675 nm). These versatile absorption and filtering performance make the proposed design highly potential for solar energy harvesting, photodetection, thermal imaging, photo-trapping, and optical communications applications.

## 1. Introduction

Metamaterials are artificially engineered or synthesized structured materials with numerous remarkable electromagnetic properties which are naturally impossible to achieve. Metamaterials have a wide range of applications in light absorption and manipulation, as they can be tuned to desired permittivity or permeability by adjusting the size and geometry of the structure [1,2]. Metamaterials are also employed in a variety of applications, including improved solar radiation to energy conversion [3], blue and ultraviolet light absorbers [4], photonic sensors [5], and direct electric power generation [6]. Metamaterials are suitable for camouflage technology [7], split ring square reflect array element [8], antennas [9,10], sensors [11,12,13,14,15,16], filters [17], physical property detection [18], and Optical MA based color converter system for optical communication [19]. The development of perfect metamaterial absorbers (MPA) for the visible and infrared spectrum has recently attracted much research attention. MPAs can be applied to manipulate light at a specific range of frequencies or wavelengths by modifying their resonance properties. Most of the MPAs intended for the visible ranges are bound by a narrow absorption spectrum, which limits their applications to solar resonators and thermal emission manipulations. Applications like solar energy converters, thermal emitters, and artificial colors with polarization and angle-insensitive properties make wideband absorbers particularly appealing.

Numerous techniques for extending the absorption band have been developed as a result of research on broadband absorbers, including periodic array [9], plasmonic ab-sorber structure [20], metamaterial transmission line absorber [21] phase-changing mate-rial-based tunable absorber [22,23] Nanoarchitectonics PMA [24], carbon nanotube ab-sorber [24]. Most conventional approaches mainly focus on a common absorption mechanism that involves coupling many resonances with a broad wavelength range excited by variously sized metallic resonators in the structures. Based on the general principle of di-polar management via structural reconstruction of kirigami-inspired meta-architecture, a transformable metamaterial has been presented in the article [25] for Adaptable Invisibility Management. They used three enantiomers with various geometries distinguished by the folding angle to exhibit reconfigurable invisibility management with a wealth of EM functions and a broad tuning range. The meta-architecture, which was created on a transparent substrate, was intended to operate between 4 and 16 GHz. Industrial technologies have difficulty producing structurally complex absorbers because of their complex structures and paresis measurements. Thus, an absorber with a compact size, broad absorption spectrum, polarization insensitive, high incident angle stable, simple fabrication, and low cost is required for optical window applications. Besides, prevention of incident light reflection is another challenge for optical absorbers. The concept of employing silicon dioxide (SiO_2_) can overcome this limitation. The SiO_2_ has the properties of suppressing incident light reflection to create a wideband absorber. Also, SiO_2_ laminating can prevent metal resonators from oxidization. In addition, Tungsten (Tu) is preferable for metal resonators due to its high intrinsic loss and impedance-matching properties with free space in optical wavelength. Tungsten can withstand high temperatures thanks to its high melting point, making it ideal for thermo-photovoltaic and other high-temperature applications [23].

This paper proposes a wideband, efficient, and straightforward novel multilayer MA for solar application in the optical and near-infrared spectrum. A layer of SiO_2_ on top works as an antireflection coating, protecting from rusting of the metal Tu layer and improving absorption. The average absorption was 92% across the optical and near-infrared spectrum, covering from 400 nm to 2400 nm wavelength. The MA is also polarization insensitive and incidence angle insensitive up to 45°. Additionally, the unit cell’s photon conversion efficiency has been calculated regarding A_AM1.5_, with the MA obtaining an A_AM1.5_ of 90.76%. A brief study of the magnetic and electric field, charge distributions, and current density have also been presented. The metamaterial absorber has the potential for certain exceptional qualities, such as simple manufacture, low cost, simultaneous wideband coverage, and perfect absorption. The excellent performance makes the proposed design attractive for applications like solar energy harvesting, photodetection, thermal imaging, photo-trapping, and optical communications.

## 2. Geometric Structure of the Absorber Unit Cell

**Figure 1:**
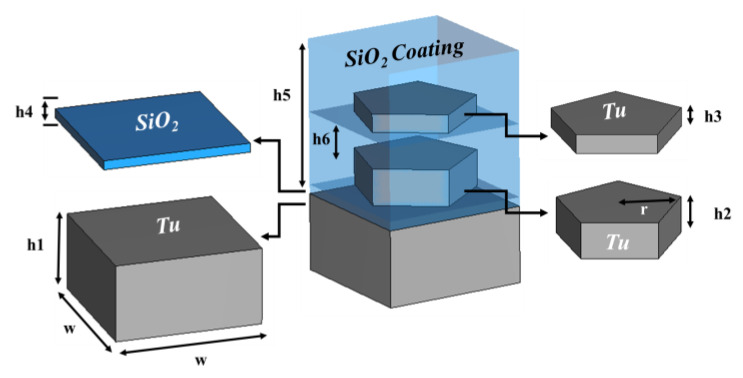
The design of the internal structure and ingredients of the layers of the unit cell.

The proposed MA structure consists of silicon dioxide (SiO_2_) and tungsten (Tu). SiO_2_ is used for the dielectric substrate layer and the antireflection coating, while Tu has been employed for the resonators and the ground layer. SiO_2_ coats the Tu resonator layers to stop incident wave reflection and layer oxidation. Figure 1 illustrates the MA unit cell’s physical structure. Despite having significant intrinsic losses, Tu was chosen for optical wavelength applications because of the excellent impedance matching with open space. Because weak transmission and reflection quality leads to increased absorption behavior [26]. SiO_2_ was chosen as a dielectric substrate material because it is lossless in the optical wavelengths. These characteristics of SiO_2_ lead to a broader absorption bandwidth and good impedance matching [26]. The nanoarchitecture design and the proposed MA characterizations were done using Computer Simulation Technology (CST). As the MA stricter is with curvature, tetrahedral mesh was used in simulation for extracting result more accurately. The estimated free space propagation impedance of the substrate’s dielectric characteristics is 377 Ω. The minimum and maximum edge lengths in this tetrahedral mesh analysis—which has a quality between 0.176825 and 0.999151—are 5.250000 nm and 141.421476 nm, respectively. The simulation was run with a mesh with a total of 12,424 tetrahedrons and an average quality of 0.781090 in order to produce the most accurate results.

The unit cell boundary condition has been applied to the x- and y-axes, and the open add space condition has been applied to the z-axis. By using Equation (1), the absorption property *A*(*ω*) was calculated. Where the reflection coefficient is *S*_11,_ and the transmission coefficient is *S*_21_.

(1) $$A\left(\omega \right)=1-{\left|S_{11}\left(\omega \right)\right|}^{2}-{\left|S_{21}\left(\omega \right)\right|}^{2}=1-R-T$$

Here *S*_21_ is zero as the thick Tu back layer blocks the transmission of the electromagnetic wave, which can be explained by skin depth calculation from Equation (2). So, Equation (1) can be written as Equation (3).

(2) $$\mathrm{skin}\;\mathrm{depth}\;\left(\delta \right)=\sqrt{\frac{\rho \omega }{\pi C\mu }}$$

(3) $$A\left(\omega \right)=1-{\left|S_{11}\left(\omega \right)\right|}^{2}=1-R$$

**Figure 2:**
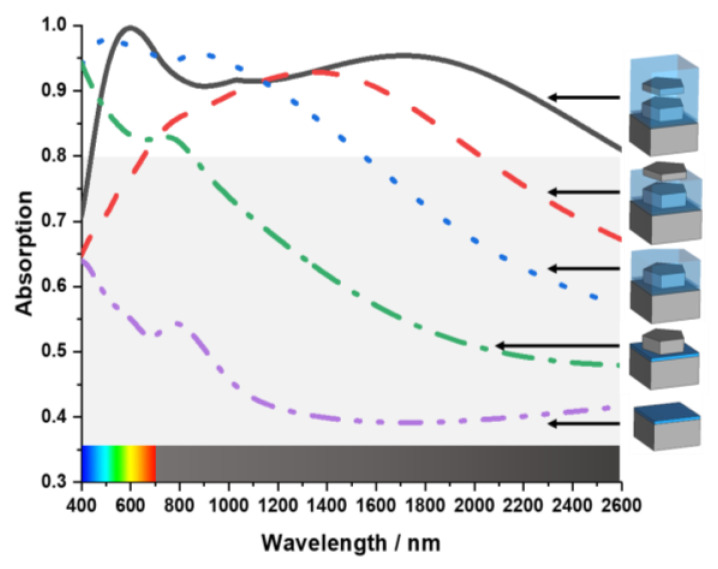
The design evaluation and its respective absorption.

In Equation (2), the resistivity (*ρ*) of Tu is 5.60 × 10^−8^ (Ωm^−1^), *ω* = 2600 nm is the maximum wavelength, and permeability μ is 1. According to Equation (2), the skin depth for this MA is 0.0125 nm, which prevents waves with a wavelength of less than 2400 nm from transmitting. The unit cell design parameters are listed in Table 1. Figure 2 shows the absorption properties of the unit cell as the design gradually evaluates toward the final structure of the MA. The MA offers very weak absorption when the unit cell only has the dielectric substrate layer of SiO_2_ and the ground. As there is no resonating element in the unit cell at this stage, all incident waves are reflected from the ground layer. After introducing the first Tu resonating layer, the MA shows a peak of 95% absorption at 400 nm wavelength. However, it is weak for the remaining wavelengths. That is mainly because the incident light is reflected from the metal resonator layer. Then a coating of SiO_2_ was introduced to prevent the incident light from reflecting. With this coating, the MA shows a 96% average absorption in the visible spectrum from 400 nm to 700 nm. But there is still a very weak absorption in the near-infrared region. A second Tu resonator layer has been added above the SiO_2_ coating to improve absorption in the near-infrared spectrum.

**Figure 3:**
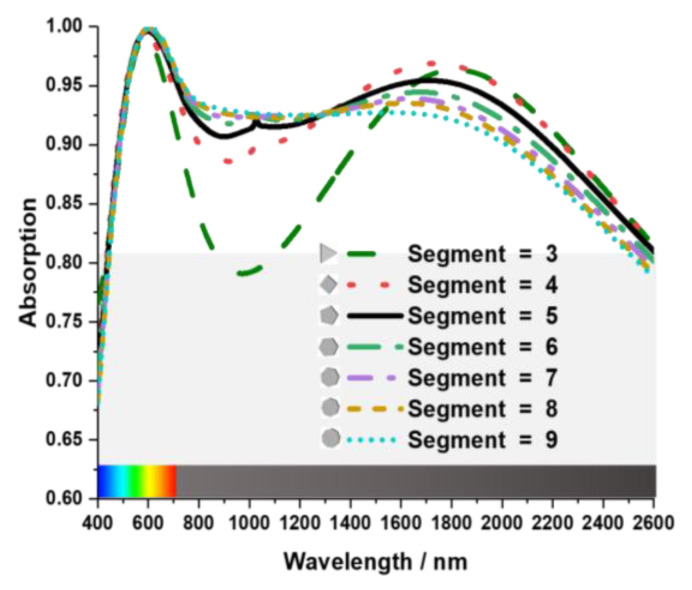
The design of the internal structure of the layers of the unit cell.

That second Tu layer effectively increased average absorption in the near-infrared spectrum but failed to achieve adequate absorption in the visible spectrum. Decreased absorption in the visible range results from reflecting light from the second metal resonating layer. Another coating of SiO_2_ was added to the final nanoarchitecture unit cell design to prevent the incident light from reflecting from the Tu layer to increase the absorption in the visible spectrum. After adding SiO_2_ coating, the MA showed a 92% average absorption from 400 nm to 2400 nm wavelength with perfect peak absorption at 600 nm wavelength. The geometrical shape of the resonating patch plays an essential role in achieving broader and higher absorption. Figure 3 shows the effect of the patch shape. When the patch had a triangular shape with three segments, it had weak absorption in 850 nm to 1700 nm wavelengths. The absorption pattern gets more consistent as the number of segments rises. However, the fabrication process becomes more challenging if the nanoarchitecture unit cell is designed with extreme precision. This study uses a patch with five segments as it has comparatively broad bandwidth and a high average absorption of 92% throughout 400 nm to 2400 nm wavelength with relatively fewer segments.

**Figure 4:**
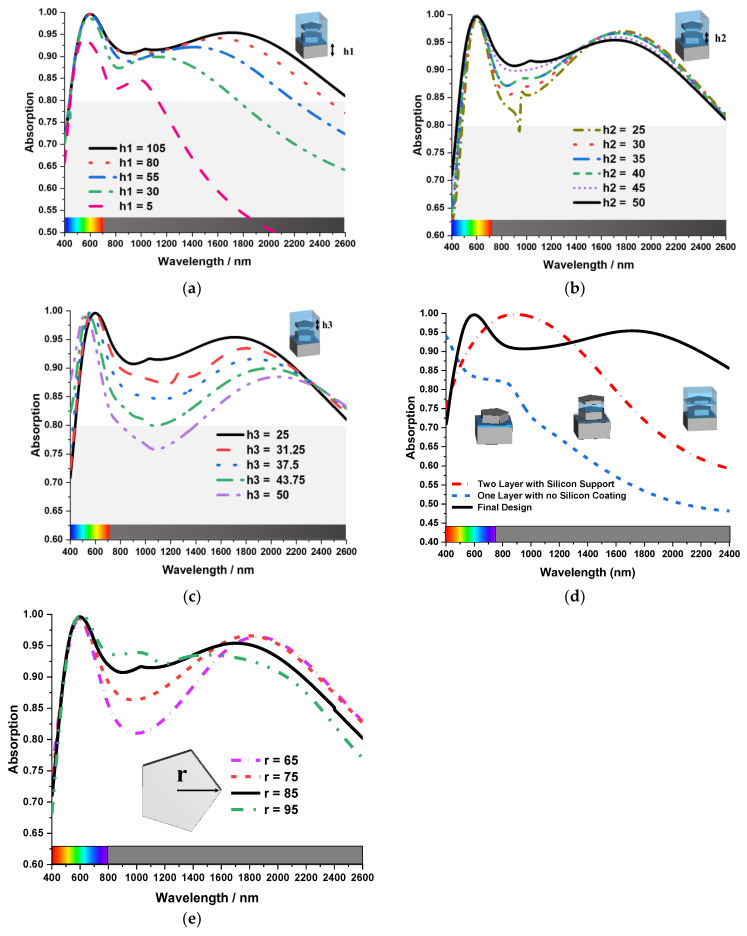
The absorption property of the unit cell for different thicknesses of (**a**) ground layer, (**b**) first resonating Tu layer, (**c**) second Tu layer, (**d**) relation between MA absorption property and SiO_2_ antireflection coating, (**e**) The influence of the Tu resonator size to the absorption property of the MA.

The absorption property of the unit cell is related to the geometry of the layers. The MA unit cell’s ground layer thickness is shown in Figure 4a by the notation h1. Absorption bandwidth and average absorption increase as h1’s value increases from 5 nm to 105 nm. The MA’s peak absorption of 93% was at 600 nm, its average absorption was 85%, and its bandwidth was 700 nm for h1 = 5 nm. As h1 increases, the peak absorption, average absorption, and bandwidth increase. In the final design, h1 is 105 nm, and it shows 92% average absorption with 2000 nm bandwidth. So, the absorption of this MA is proportional to the thickness of the ground layer. Similarly, the thickness of the first resonating Tu layer also has the same effect on the average absorption. But it does not show significant changes as the bottom layer shows in the figure. In Figure 4b, h2 represents the thickness of the ground layer of the MA unit cell. The figure shows that for a lower value of h2 average absorption decreases, and it increases as the value of h2 increases. But overall average bandwidth remains the same for different values of h2. 

In Figure 4c, the thickness of the second Tu layer has the opposite effect on the average absorption. Here h3 is the thickness of the second Tu layer. If the thickness of the second Tu layer (h3) increases, the average absorption decreases. When h3 decreases, the opposite occurs. However, the overall average bandwidth for h3 is unchanged. The relationship between the MA absorption property and the SiO_2_ antireflection layer is depicted in Figure 4d. The SiO_2_ anti-reflection layer primarily changes the absorption bandwidth. There is a 99.6%-peck absorption in all three cases of no SiO_2_ coating, with a SiO_2_ support layer in between two Tu resonating layers and the final design with full SiO2 anti-reflection coating. But full SiO_2_ anti-reflection coating structure has achieved a wide bandwidth of 2000 nm. Based on the theory presented in the article [27], a single layer of anti-reflective coatings of glass substrates should have a thickness of 1/4 of the incident wavelength and a refractive index of ~1.22 for reflective light from the air to film interface and from film-substrate interface together. SiO_2_ is transparent from 160 nm to 3000 nm wavelength and its corresponding refractive index of SiO_2_ is 1.55 to 1.40, which is closer to the desired refractive index. The thickness of the SiO_2_ layer in the proposed MA structure is 100 nm and hence, the SiO_2_ layer can be used as an anti-reflective coating from wavelengths above 400 nm.

In the Figure 4e, the influence of the Tu resonator size to the absorption property of the MA has been presented. Absorption bandwidth and average absorption increase as the Tu resonator (r) size changes from 65 nm to 95 nm. The MA’s absorption was less than 85% from 800 nm to 1300 nm (the beginning near-infrared wavelengths) when r was 65 nm. As the resonator size (r) increases to 75 nm, the absorption in the beginning near infrared wavelengths increases, but overall bandwidth remains the same. When the value of r was 85 nm and 95, the absorption increases more than 90% in the 800 nm to 1300 nm wavelengths range, but the overall bandwidth decreases. In the proposed MA, the value of r was 85 nm, as it achieved average absorption of 92% with 2000 nm bandwidth.

## 3. Results Analysis

### 3.1. Unit Cell Absorption Characteristics

**Figure 5:**
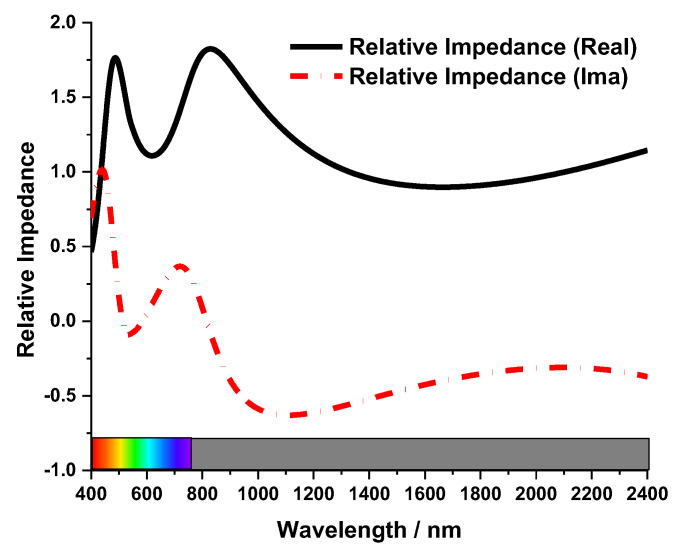
The relative impedance of the MA.

Impedance matching plays a critical factor in MA absorption characteristics. The equation for relative impedance for the MA is given in Equation (4). [28] The relative impedance, permeability, and permittivity of the MA are represented by *Z*, *μ* (*μ* = *μ_r_μ*_0_), and *ε* (*ε* = *ε_r_ε*_0_) respectively. The relative impedance of the MA is presented in Figure 5. The mechanism for wideband operation can be explained by the absorber’s normalized impedance. When the absorber’s normalized impedance (*Z*) matches with the free-space impedance (*Z* = *Z*_0_ = 1), the perfect absorption has been attained. From the Equation (5), we can say, the closer the imaginary part is to zero, and the closer the real part is to one, the higher the absorption of the MMA. The Figure 5 shows that the impedance of the MA matches with the free space impedance for the wavelength 400 nm to 2400 nm.

(4) $$Z=\sqrt{{\left(1+S_{11}\right)}^{2}-\frac{S_{21}^{2}}{{\left(1-S_{11}\right)}^{2}}-S_{21}^{2}}=\frac{\sqrt{\mu /\varepsilon }}{Z_{0}}=\sqrt{\mu_{r}/\varepsilon_{r}}$$

(5) $$A_{TM,TE}=1-{\left|\frac{Z-Z_{0}}{Z+Z_{0}}\right|}^{2}=1-{\left|\frac{\sqrt{\mu_{r}}-\sqrt{\varepsilon_{r}}}{\sqrt{\mu_{r}}+\sqrt{\varepsilon_{r}}}\right|}^{2}$$

**Figure 6:**
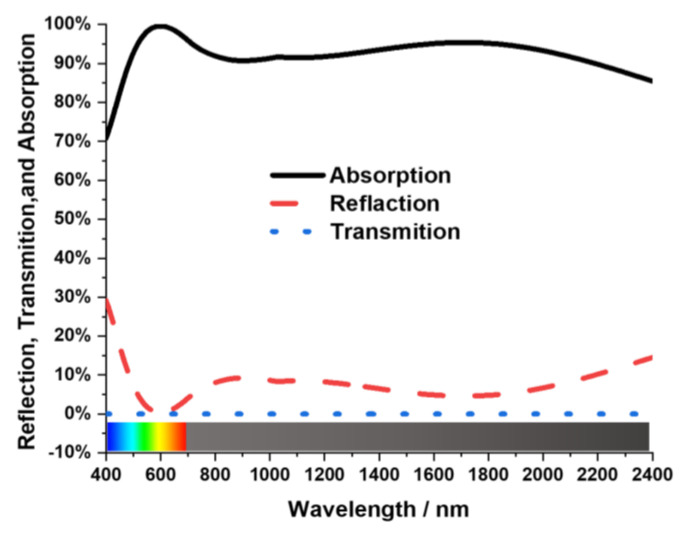
The reflectance (red), transmittance (blue), and absorption (black) characteristics of the MA.

Here, *μ_r_* represents the relative permeability and permittivity is represented by *ε_r_* in Equation (4). For free space, those are *μ*_0_ and *ε*_0_. Electromagnetic waves cannot transmit through the Tu back layer, so transmission is considered zero. Absorption, which is denoted as A, can be derived from A = 1 − R. Here R is reflectance. The reflectance, absorption and transmittance characteristics of the MA are given in Figure 6. The proposed MA’s symmetrical design gives a unique absorption curve. An absorption above 99% appeared from 565 nm to 635 nm with a 99.6% peak absorption at 600 nm wavelength. The MA obtained a 92% average absorption bandwidth of 2000 nm for the visible and near-infrared spectrum. The near-unity absorption feature of the suggested MA is advantageous for the visible optical wavelengths of solar energy harvesting, sensors, micro-imaging technologies and stealth technology [29].

### 3.2. Study of TE and TM Polarization

**Figure 7:**
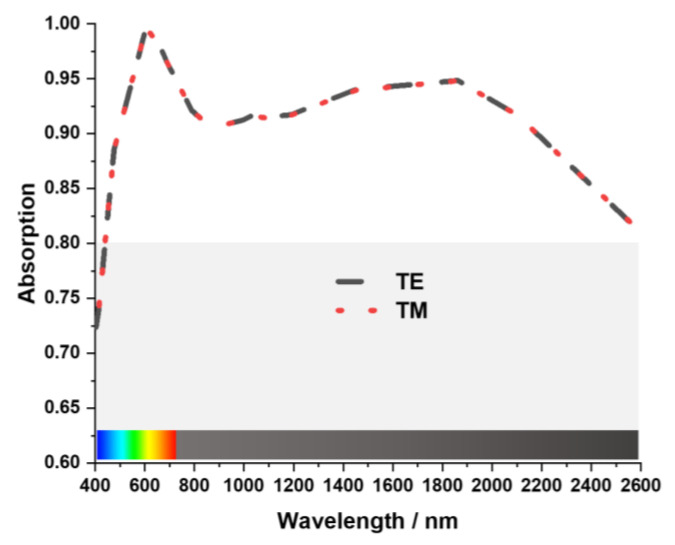
Absorption characteristics of TE and TM mode.

Transverse Electric (TE) and Transverse Magnetic (TM) modes are utilized to characterize the properties of the MA to explain its absorption. In TE Mode incident wave propagates in the direction of the z-axis. The propagation of the magnetic field vector is along the z-axis. The magnetic and electric field vector is along the y and x-axis, respectively. In TM Mode incident wave propagates in the direction of the z-axis. Also, the electric field vector propagates along the z-axis. The magnetic and electric field vectors are along the x and y-axis. Figure 7 shows that the MA exhibit identical absorption characteristics for both TE and TM modes throughout the resonating bandwidth. These characteristics indicate that the structure proposed in this work can be employed as an ideal solar energy harvester, sensors, stealth technologies, and micro-imaging equipment. 

### 3.3. Co-Polarization and Cross-Polarization

**Figure 8:**
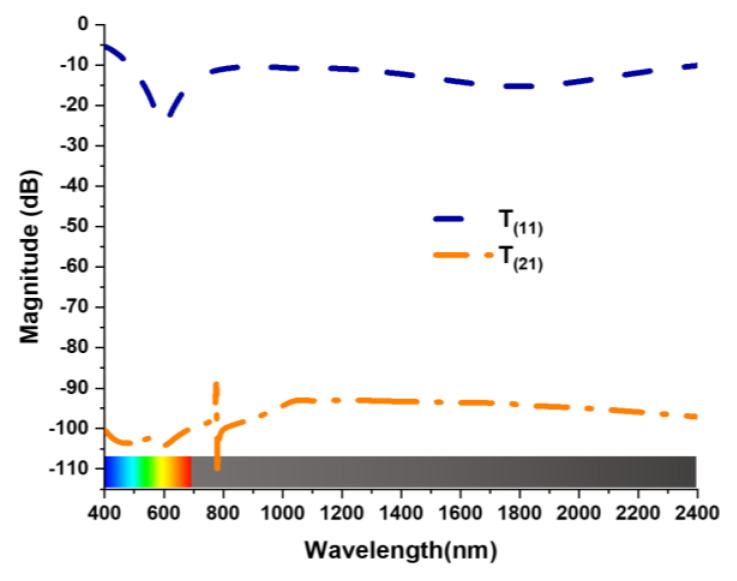
Co-polarization and cross-polarization.

This MA is not a polarization converter. The PCR value of the proposed design has been calculated to verify the absorption of the MA. Co-polarization and cross-polarization *T*_(11)_ and *T*_(21)_ of the oblique wave are illustrated in Figure 8. This co-polarization and cross-polarization of the incident wave are achieved because of the symmetric in the MA structure. *PCR* value was calculated by Equation (6) [30].

(6) $$PCR=T_{11}^{2}/\left(T_{21}^{2}+T_{11}^{2}\right)$$

**Figure 9:**
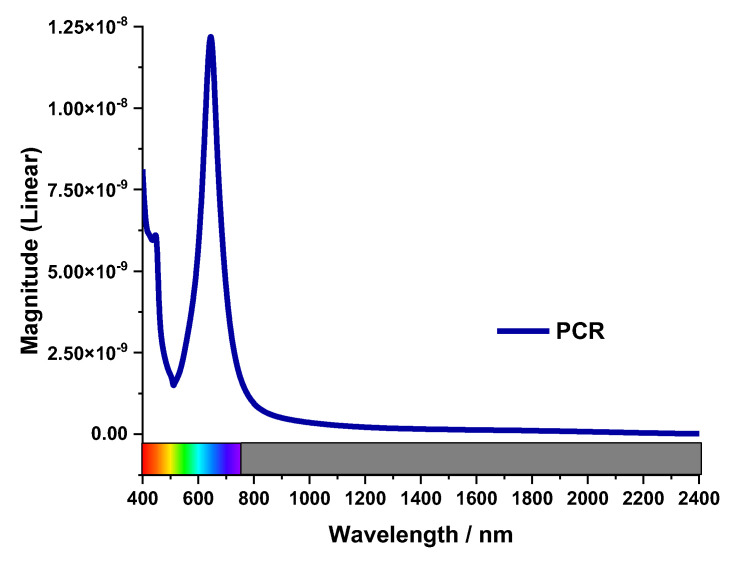
PCR value.

The polarization Conversion Ratio was negligible as cross-polarization *T*_(21)_ < −90 dB. The value of PCR in Figure 9 verifies that the MA is an absorber, not a polarization converter.

### 3.4. Metamaterial Property

**Figure 10:**
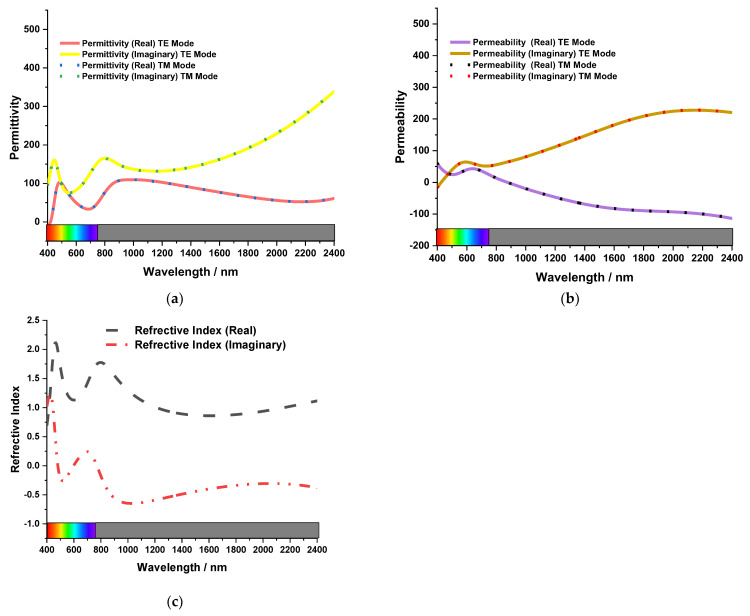
The MA’s (**a**) Permittivity and (**b**) Permeability (**c**) Refractive Index.

The MA’s Imaginary part of permittivity and permeability for both TE and TM modes and the refractive index is given in Figure 10. The effective refractive index (*η_r_*) is calculated by Equation (7). Where k = 2πλ/c, c is the velocity of light, *S*_11_ is the reflection coefficient, *S*_21_ is the transmission coefficient, n is the integer number and d is the substrate thickness. Equations (8) and (9) are also used to retrieve the relative permittivity (*ε_r_*) and relative permeability (*μ_r_*), where *Z_r_* is the relative impedance.

(7) $$\eta_{r}=\pm \frac{1}{kd}\cos^{-1}[\frac{1}{2S_{11}}(1-S_{11}^{2}+S_{21}^{2})+2n\pi ]$$

(8) $$\varepsilon_{r}=\frac{\eta_{r}}{Z_{r}}$$

(9) $$\mu_{r}=\eta_{r}Z_{r}$$

The imaginary part of permittivity is positive in the figure from 400 nm to 2400 nm wavelength. Also, the imaginary part of permeability is negative between 800 and 2400 nanometers. On the other hand, the real refractive index is positive in the resonating wavelengths. The effective EM parameters also cause the magnetic and electric resonance because it can be seen that permittivity and permeability were changed close to resonance peaks, improving the absorbing strength and boosting broadband absorption.

### 3.5. Polarization and Oblique Incident Angle Sensitivity of the MA

**Figure 11:**
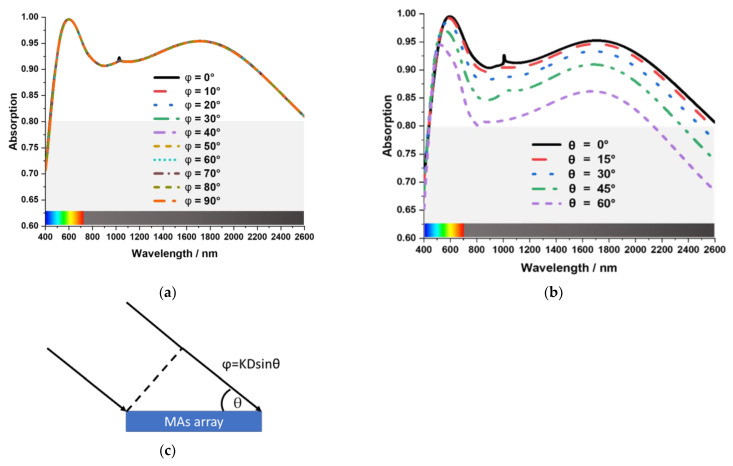
(**a**) The polarization insensitivity. (**b**) The oblique incident angle insensitivity (**c**) phase shift at the oblique incident angle.

The proposed absorber’s adaptive capacity for various polarization angles was analyzed for different polarization incident angles of EM waves. Figure 11a compares absorption for different polarization angles where φ is the polarization angle. In TE Mode, the incident wave and the magnetic field vector propagates in the direction of the z-axis. The magnetic and the electric field vector are along the y and x-axis. The MA is polarization insensitive, as the MA exhibits no significant variation in the absorption properties for polarization angles (φ) up to 90°. In Figure 11b, a comparison of absorption characteristics for different oblique incident angles has been presented. The oblique incident angle is given by θ, which is along the z-axis of the MA structure. From the figure, it can be seen that the absorption characteristics of the MA were found stable up to θ = 45° oblique incident angle as the average absorption is above 80%. The MA attained polarization insensitivity primarily because of its symmetrical resonators. Another reason the MA achieves significant incidence angle insensitivity is the high critical angle of reflection of SiO_2_. The high critical angle of reflection of SiO_2_ is 55° in the infrared optical spectrum [31]. As a result, the MA shows high absorption, almost up to 45°. As the incident angle increases more than 55°, absorption decreases rapidly. Moreover, the compact MA structure achieved the oblique incident angle stability due to the lower phase shift at the oblique incident angle. In Figure 11c at MAs array structure, for the oblique incident angle θ, the face shift φ proportionally depends on the periodicity D of the unit cell. This phase difference caused the MAs impedance mismatch with free space, reducing the absorption at a higher θ value larger than 45°.

### 3.6. Mechanical Stress

**Figure 12:**
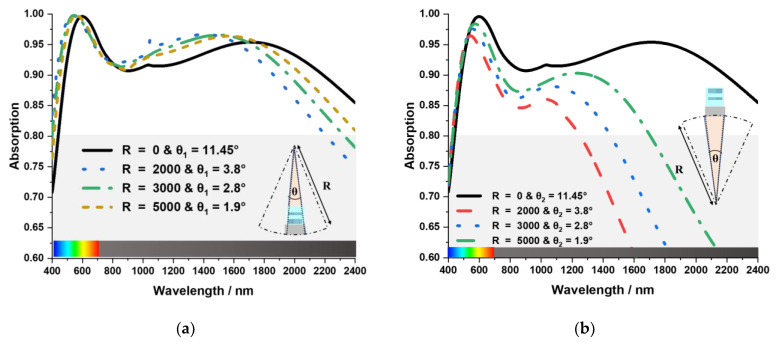
The MA is bending (**a**) toward the inside (**b**) toward the outside.

The MA can get curved under physical stress as the structure is a very small-scale nanoarchitecture. The absorption characteristics of the MA has been investigated in various banding situation. Figure 12 shows the absorption behaviour of the MA as it bends towards the inside (Concave Bending). Here R is the bending diameter, and θ_1_ is the acquired central angle for a single unit cell. Figure 12b shows the absorption behaviour of the MA as it bends towards the outside (Convex Bending). Here θ_2_ is the acquired central angle for a single unit cell. The proposed MA’s absorption behaviour is more stable when it bends towards the inside. Overall bandwidth and peak absorption remain almost the same. But its losses its stability as it bends towards the outside. Also, overall bandwidth and peak absorption decrease rapidly.

### 3.7. Performance Evaluation for Solar Application

A solar thermos photovoltaic (STPV) system needs three main components to work effectively. First component is broadband absorber that can absorb the entire solar spectrum, second one is narrowband emitter that converts absorbed energy from the absorber into heat and photons in a specific range above the PV cell’s bandgap, and the last component is low bandgap PV cell to generate electron-hole pairs without thermalization losses. Metamaterials can be used to create the narrowband emitter and broadband absorber [32]. The efficiency of the emitter and absorber is given in Equation (10). *A_ab_* and *A_em_* represent the area of the absorber and emitter. The ratio of the area is *β*. *P_sol_*, *P_ab_* and *P_em_* is Thermal irradiance of the Sun, absorber, and emitter. *P_sol_* can be defined as input power. *P_ab_*, *P_em_* and *P_sol_* is described in Equations (11)–(13). T and N_s_ is the temperature in kelvin and solar concentration, respectively. *φ* is the polarization angle, *θ* is the incident angle, *ε_em_* is emitter emittance, *ε_am_* is absorber emittance, *λ* is the wavelength, *I_BR_* is the equilibrium temperature blackbody radiation, and *I_sol_* is solar radiation spectrum. *P_ab_* at *T* temperature is given at Equation (14).

(10) $$\eta_{int}=\frac{A_{em}P_{em}}{A_{ab}P_{sol}}=\beta \frac{P_{em}}{P_{sol}}$$

(11) $$P_{ab}\left(T\right)=\overset{2\pi }{\underset{0}{\int }}d\omega \overset{\frac{\pi }{2}}{\underset{0}{\int }}\sin\left(\theta \right)\cos\left(\theta \right)d\theta \int_{0}^{\infty }\varepsilon_{ab}\left(\lambda ,\theta ,\varphi \right)I_{B}\left(\lambda ,T\right)d\lambda$$

(12) $$P_{em}\left(T\right)=\overset{2\pi }{\underset{0}{\int }}d\omega \overset{\frac{\pi }{2}}{\underset{0}{\int }}\sin\left(\theta \right)\cos\left(\theta \right)d\theta \int_{0}^{\infty }\varepsilon_{em}\left(\lambda ,\theta ,\varphi \right)I_{B}\left(\lambda ,T\right)d\lambda$$

(13) $$P_{sol}\left(T\right)=\overset{2\pi }{\underset{0}{\int }}d\omega \overset{\theta_{c}}{\underset{0}{\int }}\sin\left(\theta \right)\cos\left(\theta \right)d\theta \int_{0}^{\infty }\varepsilon_{ab}\left(\lambda ,\theta ,\varphi \right)I_{sol\left(\frac{B}{A.M\;1.5}\right)}\left(\lambda \right)d\lambda$$

(14) $$P_{ab}\left(T\right)=P_{sol}\left(N_{s}\right)-\beta P_{em}\left(T\right)$$

The proposed MA can be used as a broadband absorber that absorbs the solar spectrum completely. Using Equation (14) in (10), the equation Equation (15) can be attained, which is independent of the emitter’s thermal irradiance, making the absorber independent from the emitter.

(15) $$\eta_{int}=\frac{P_{sol}-P_{ab}}{P_{sol}}=1-\frac{P_{ab}}{P_{sol}}$$

**Figure 13:**
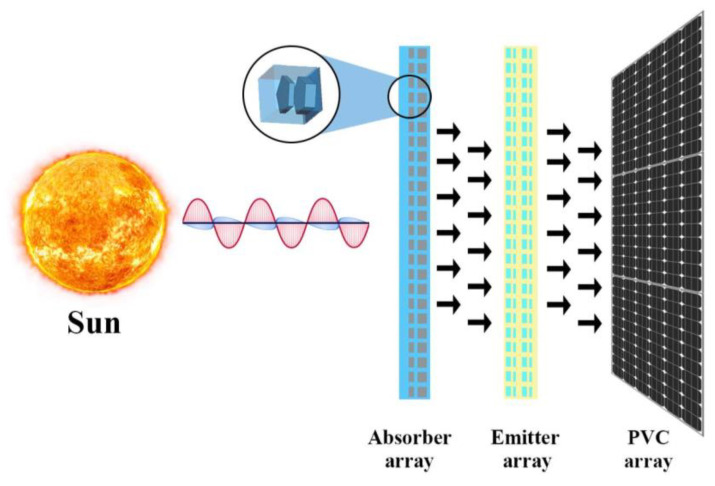
MA-based STPV system design.

Figure 13 shows the position of MA in the STPV system. Variations of the absorber unit cell structure can result in different absorption in the solar spectrum. The solar spectrum universal characterization method (*A_AM_*_1.5_) has been used to characterize terrestrial power generation of the MA universally. This method indicates the MA’s feasibility for solar cell applications. Also, photon conversion is proportional to conversion efficiency values, which results in significant electron mobility. Given below is the expression mentioned in [29] for *A_AM_*_1.5_ calculation.

(16) $$A_{A.M1.5}=\frac{\int_{W_{1}}^{W_{2}}A\left(W\right)N\left(W\right)dW}{\int_{W_{1}}^{W_{2}}N\left(W\right)dW}$$

Here in Equation (16), W is the wavelength of light, *A*(*W*) is the absorption of the proposed design, and *N* (*W*) is the allocation of photon numbers in the *AM*1.5 solar illumination, a function of wavelength and is expressed as

(17) $$N\left(w\right)=\frac{S\left(w\right)}{E\left(w\right)}$$

In Equation (17), *E*(*W*) is the energy of photon and *S*(*W*) is the solar spectral irradiance. ASTM G173-03 reference solar spectral irradiance (global tilt) has been used to calculate *N*(*W*).

After deriving *N*(*W*)and *A*(*W*), the *A_AM_*_1.5_ has been calculated using Equation (16). The final structure of the MA unit cell obtained 90.76% *A_AM_*_1.5_. High value of *A_AM_*_1.5_ is an indicator of high solar cell photon conversion efficiency [33]. So, the calculated *A_AM_*_1.5_ implies that efficiency of soler cell photon conversion would be high by considering the proposed meta surface design.

**Figure 14:**
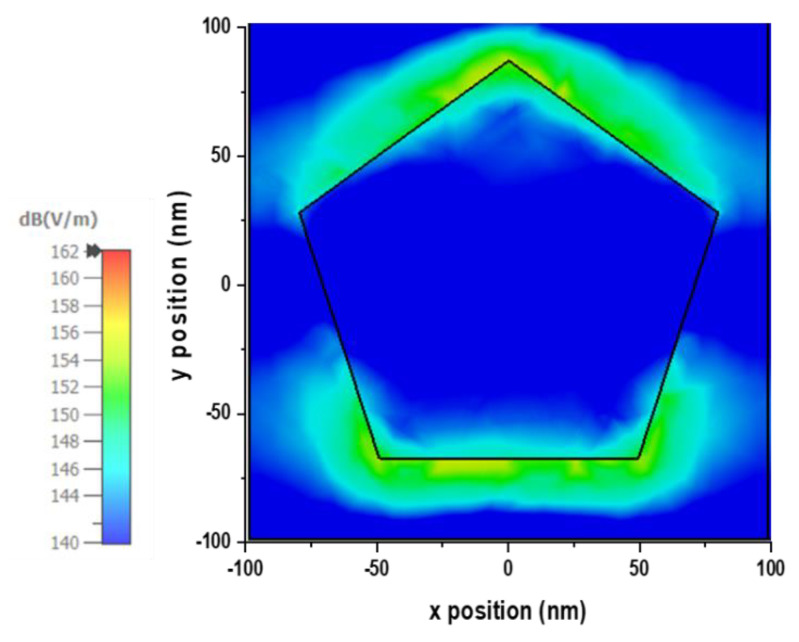
Metal nanoparticles show an intense electric-field close to the surface.

A solar absorber must be optically thick to allow near-unity light absorption and photocarrier current collection [34]. That’s why thickness of the traditional wafer-based solar cells is typically ranged from 180 to 300 μm, which increase the overall size of the cell and makes the structure oblique angle sensitive. Nanostructured metallic particles (Plasmonic structures) absorbers can address those conflicting demands. A thin absorber like the proposed plasmonic MA sandwiched in semiconductor material can take advantage of the strong local electrical field enhancement around the metal nanoparticles to increase absorption. In this sandwich structure, nanoparticles act as an antenna to store incident energy as localized surface plasmon mode for the sunlight. Besides, the photocarriers must be generated near to the collection junction area and the carrier diffusion lengths should be smaller to achieve higher energy conversion efficiency. Also, the absorption rate in the semiconductor must be larger than the reciprocal of the typical plasmon decay time, which can be seen for the proposed MA in the Figure 14. In the Figure 14, the metal nanoparticles show an intense electrical field close to the surface for TE wave propagation for with a wavelength λ = 800 nm in normal incident angle.

### 3.8. Performance Evaluation for Optical Communications

The proposed nanostructure can be used for filtering applications at optical communication by modifying ground layer of the plasmonic nano structure. Optical fibre communications use the spectral region from 800 to 1675 nm. For usage in intermediate-range and long-distance optical fibre communications in the 1260–1675 nm range, the International Telecommunications Union (ITU) has designated six spectral bands [35]. The regions are identified by the letters O, E, S, C, L, and U, as shown in Table 2.

**Figure 15:**
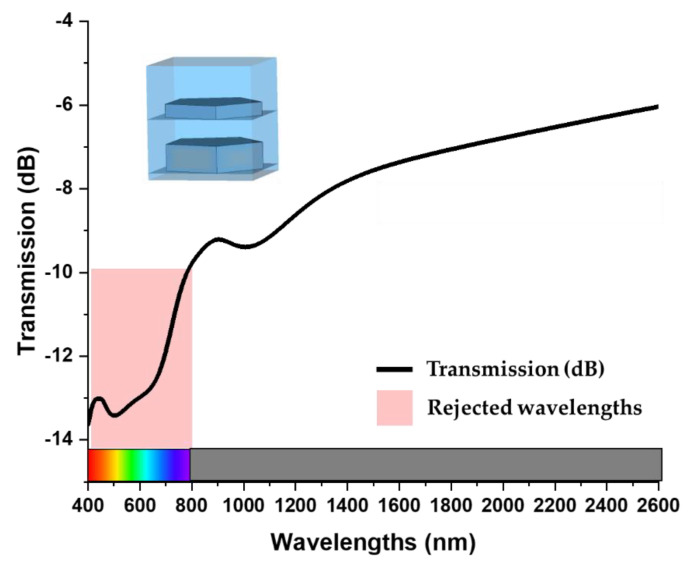
The transmittance characteristics of the nanostructure.

To evaluate the performance of the nanostructure as a filter, the Tu ground layer has been removed from the MA structure. As a result, the EM waves starts to transmit through the nanostructure. From the Figure 15, it can be seen that the nanostructure blocks the wavelengths of visible band and allows the optical bands to transmit. The wavelengths lower then 800 nm are experiencing high transmission loss while the optical communication bands are transmitting with a relatively lower transmission loss. So, the proposed nanostructure can be used as a filter for optical communication to filter out noises from visible wavelengths.

### 3.9. Electric and Magnetic Field

**Figure 16:**
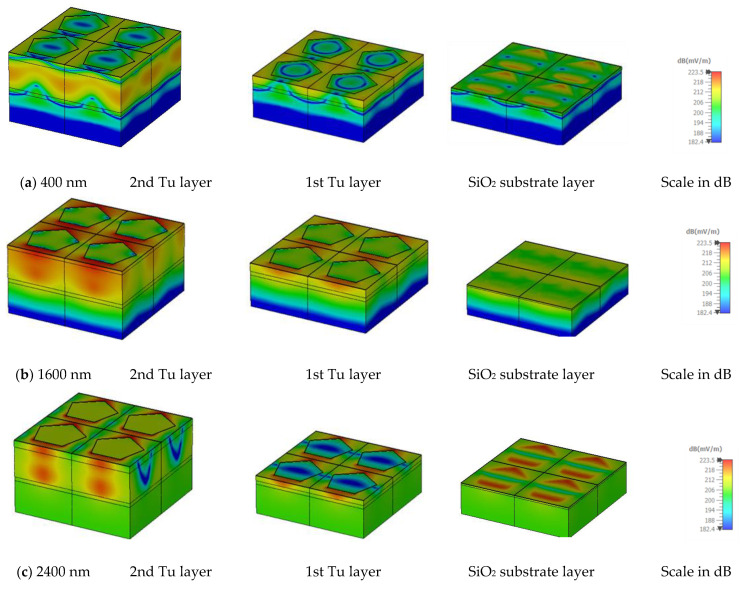
E-field distribution at different layers in (**a**) 400 nm, (**b**) 1600 nm, and (**c**) 2400 nm wavelength.

**Figure 17:**
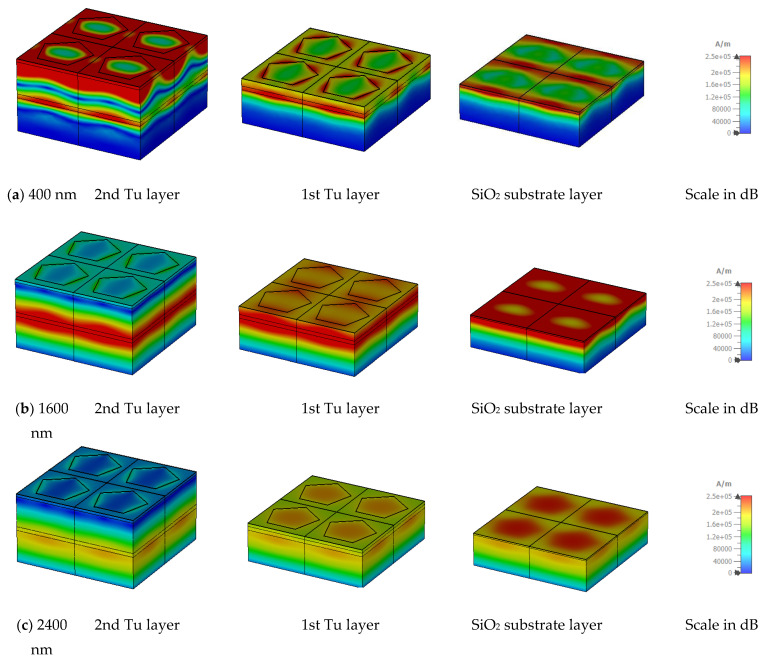
H-field distribution at different layers in (**a**) 400 nm, (**b**) 1600 nm, and (**c**) 2400 nm wavelength.

Figure 16 and Figure 17 illustrate the e-field (mV/m) and h-field (A/m) in MA for different layers at different wavelengths. All measurements have been done in TE mode at an oblique angle θ = 0°. The electromagnetic behaviour is related to the absorption mechanism. The electric field (e-field) and magnetic field (h-field) distribution at 400 nm, 1600 nm, and 2400 nm wavelengths in TE mode are presented. The correlation of MA characteristics and electromagnetic performance can be understood by Equations (18) and (19).

(18) $$B_{avg}=\mu_{eff}\mu_{0}H_{avg}$$

(19) $$D_{avg}=\varepsilon_{eff}\varepsilon_{0}E_{avg}$$

Here *ε*_0_ and *μ*_0_ is permittivity and permeability of free space, respectively. Effective permittivity and permeability is demoted by *ε_eff_* and *μ_eff_*. The mean electric and magnetic field intensity is *E_avg_* and *H_avg_*. *B_avg_* and *D_avg_* are mean magnetic and electric flux density. Integral Maxwell’s equation for *B_avg_* and *D_avg_* can be written as Equations (20) and (21).

(20) $$\overset{\;}{\underset{c}{\int }}E.dI=0-\frac{\partial }{\partial t}\overset{\;}{\underset{s}{\iint }}B.dS$$

(21) $$\overset{\;}{\underset{c}{\int }}H.dI=0-\frac{\partial }{\partial t}\overset{\;}{\underset{s}{\iint }}D.dS$$

When the magnetic field exhibits non-uniform fluctuation that changes quickly after an electric field wave passes across it, the integral function is calculated simultaneously. In a uniform field distribution, permittivity typically reaches unity; however, in this scenario, Figure 16 and Figure 17 show that the magnetic field and electric field are asymmetrical and non-uniform, and the consequent EM wave propagation is also non-uniform. Figure 16 and Figure 17 clearly demonstrate that the electric field and the magnetic field depend on the physical shape of the metal resonator and the dielectric layer. The penetrating wave excites mainly at the Tu resonators’ outer section. As the wavelengths increase, the excitation area and depth increase for both the electric and magnetic field intensity. In lower wavelengths, the excitation pattern of SiO_2_ is almost the same for both E and H fields. At 450 nm wavelength, E and H field intensity is comparatively strong at the corners of the Tu and in the surrounding SiO_2_. But in the higher wavelengths, E and H field intensity is different. At 1600 nm wavelength, E field intensity is strong in the middle of the Tu layers, while H field intensity is strong at the corners of the Tu layers. In the case of 2400 nm wavelength, if the E field intensity is strong in the vertical corners of the Tu layer, then H field intensity is strong at the horizontal corners of the Tu layers.

### 3.10. Comparison

A comparison of the proposed MA with some of the recent works has been presented in Table 3. However, only a few of them are applicable for solar applications. Some of them have a complex multilayer structure and makes difficulties during fabrication. Besides, these works are suffering from relatively low angular stability reduced the solar absorber efficiency. On the other hand, the proposed MA achieved wide angular stability and high photon conversion efficiency for solar applications with a comparatively simple structure. The operating wavelength, average absorption, number of layers, angular stability, material choice, dimension, use of anti-reflective layer, and A_AM1.5_ have been compared in Table 3. The proposed compact broadband MA offers an average absorption of 92% and a near-unity peak absorption of 99.6%. at 600 nm with polarization insensitivity. The anti-reflective layer on the unit cell plays a vital role for maintaining broadband absorption. The proposed MA obtained 90.76% A_AM1.5_ photon conversion efficiency, making the proposed pentagonal multilayered MA attractive for energy-harvesting applications.

## 4. Conclusions

This article proposes a pentagonal multilayered wideband metamaterial absorber for the visible and near-infrared region. The overall diminution of the MA unit cell is 0.5λ × 0.5λ × 0.8λ. The proposed MMA has strong absorptivity in the visible and near-infrared regions, with 92% average absorption throughout 400 nm to 2400 nm wavelength. The MA attained 2000 nm bandwidth with a peak absorption of 99.6% at 598 nm. The SiO_2_ coating on Tu resonating layers plays a vital role in incident light reflection prevention. The proposed metamaterial absorber features consistent oblique incidence angle stability for both TM and TE polarization and polarization-independent characteristics. In order to interpret the absorption mechanism, the metamaterial property, magnetic field, electric field, and surface current distribution are investigated. Finally, the MA obtained 90.76% A_AM1.5_ photon conversion efficiency, thus making the MA applicable in solar energy harvesting applications. Therefore, the proposed MA has excellent potential in various applications, including thermal emitters, infrared imaging, photodetectors and energy harvesters.

## Figures and Tables

**Table 1:** Structural parameters of the MA unit cell.

Parameter Symbol	Value (nm)	Parameter Symbol	Value (nm)
h1	105	h5	200
h2	50	h6	50
h3	25	w	200
h4	12.5	r	85

**Table 2:** The ITU designated six spectral bands for optical communication.

Band	Wavelengths (nm)	Band	Wavelengths (nm)
O	1260 to 1360	C	1530 to 1565
E	1360 to 1460	L	1565 to 1625
S	1460 to 1530	U	1625 to 1675

**Table 3:** Comparison of the proposed MA with the recent design.

Ref	WL (in nm)	Operating Frequency Band (THz)	Average Absorption	Number of Layers	Polarization Independency and Angular Stability	Used Materials	Dimensions (length × width × height)	Anti-reflective Layer	A_AM1.5_
[32]	200–1300	230–1500	87%	3	N/A	Cr, SiO_2_	3λ × 3λ × 1.3λ	No	88.50%
[22]	580–1000	300–516	95.2%	2	Independent, θ = 30°	Ge_2_, Sd_2_, Te_5_	0.4λ × 0.4λ × 0.21λ	No	N/A
[36]	450–650	460–665	91.82%	3	Independent, θ = 40°	Tu,Si	1.8λ × 1.8λ × 0.4λ	No	N/A
[26]	280–700	428–1070	90%	3	Independent, θ = ≤ 30°	Tungsten, SiO_2_	2λ × 2λ × 0.4λ	No	N/A
[37]	450–3000	100–666	90%	3	Independent, N/A	Fe,Si,Au	0.6λ ×0.6λ × 2λ	No	N/A
[38]	435–615	450–650	–	4	Dependent, θ = 30°	rs43, SiO_2_,Al	1λ × 1λ × 1.5λ	No	N/A
[39]	500–1700	175–600	82%	6	Independent, θ = ≤ 30°	SiO_2_, Ti, MgF2, Au, TiO_2_	0.6λ × 0.6λ × 1λ	No	N/A
[31]	300–700	428–750	92.2%	3	Independent, θ = ≤ 45°	Tungsten, SiO_2_	3.2 λ × 3.2λ × 0.77λ	No	N/A
Proposed MA	400–2400	125–750	92%	3	Independent, θ = ≤ 45°	Tungsten, SiO_2_	0.5λ × 0.5λ × 0.5λ	Yes	90.76%

## Data Availability

All data are fully available without restriction.

## Abbreviations

Metamaterial Absorber (MA); Tungsten (Tu); Gold (Au); Silicon Di-Oxide (SiO_2_); Atmospheric; Transparency Window (ATW); Perfect Metamaterial Absorber (PMA); Specific Absorption Rate (SAR); Transverse Magnetic (TM); Transverse Electric (TE); Refractive Index (RI); Antireflection (AR); Peak Absorption Frequency (PAF); Computer Simulation Technology (CST); Polarization Conversion Ratio (PCR)
